# High-Throughput, Kingdom-Wide Prediction and Annotation of Bacterial Non-Coding RNAs

**DOI:** 10.1371/journal.pone.0003197

**Published:** 2008-09-12

**Authors:** Jonathan Livny, Hidayat Teonadi, Miron Livny, Matthew K. Waldor

**Affiliations:** 1 Channing Laboratories, Brigham and Women's Hospital, Harvard Medical School, Boston, Massachusetts, United States of America; 2 Computer Sciences Department, University of Wisconsin-Madison, Madison, Wisconsin, United States of America; 3 Howard Hughes Medical Institute, Channing Laboratories, Brigham and Women's Hospital, Harvard Medical School, Boston, Massachusetts, United States of America; Centre for DNA Fingerprinting and Diagnostics, India

## Abstract

**Background:**

Diverse bacterial genomes encode numerous small non-coding RNAs (sRNAs) that regulate myriad biological processes. While bioinformatic algorithms have proven effective in identifying sRNA-encoding loci, the lack of tools and infrastructure with which to execute these computationally demanding algorithms has limited their utilization. Genome-wide predictions of sRNA-encoding genes have been conducted in less than 3% of all sequenced bacterial strains, leading to critical gaps in current annotations. The relative paucity of genome-wide sRNA prediction represents a critical gap in current annotations of bacterial genomes and has limited examination of larger issues in sRNA biology, such as sRNA evolution.

**Methodology/Principal Findings:**

We have developed and deployed SIPHT, a high throughput computational tool that utilizes workflow management and distributed computing to effectively conduct kingdom-wide predictions and annotations of intergenic sRNA-encoding genes. Candidate sRNA-encoding loci are identified based on the presence of putative Rho-independent terminators downstream of conserved intergenic sequences, and each locus is annotated for several features, including conservation in other species, association with one of several transcription factor binding sites and homology to any of over 300 previously identified sRNAs and cis-regulatory RNA elements. Using SIPHT, we conducted searches for putative sRNA-encoding genes in all 932 bacterial replicons in the NCBI database. These searches yielded nearly 60% of previously confirmed sRNAs, hundreds of previously annotated cis-encoded regulatory RNA elements such as riboswitches, and over 45,000 novel candidate intergenic loci.

**Conclusions/Significance:**

Candidate loci were identified across all branches of the bacterial evolutionary tree, suggesting a central and ubiquitous role for RNA-mediated regulation among bacterial species. Annotation of candidate loci by SIPHT provides clues into the potential biological function of thousands of previously confirmed and candidate regulatory RNAs and affords new insights into the evolution of bacterial riboregulation.

## Introduction

sRNAs are small (typically 100–300 nucleotides in length), non-coding bacterial RNAs that regulate gene expression, usually by interacting with specific mRNA targets to modulate message stability and/or accessibility to the translation machinery [1]. sRNAs have been shown to regulate a wide variety of cellular processes including secretion, quorum sensing, stress responses, and virulence [1], [2]. In nearly all cases, genes encoding sRNAs are located far from genes encoding their mRNA targets and sRNA:mRNA hybridization occurs over relatively short regions of imperfect complimentarity. Due to this limited sRNA:mRNA complimentarity, predicting the regulatory role of confirmed sRNAs through bioinformatic identification of their mRNA targets has proven very difficult. In recent years, more than 200 sRNAs have been identified in several bacterial species including *Escherichia coli*, *Vibrio cholerae, Pseudomonas aeruginosa, Bacillus subtilis, Listeria monocytogenes*, and several cyanobacterial species [3]. While the diversity of species known to encode sRNAs suggests that these riboregulators are common to most if not all branches of the bacterial evolutionary tree, genome-wide predictions of sRNA-encoding genes have been conducted in only 19 of the over 550 sequenced bacterial strains [3]. Moreover, the biological activities of only a small minority of confirmed sRNAs are currently known. The relative paucity of sRNA predictions and functional annotations represents a significant gap in our understanding of bacterial genomes, transcriptomes, and regulatory pathways.

The lack of genome-wide annotations for sRNA-encoding genes compared to those for genes encoding proteins, tRNAs, and rRNAs is due in large part to the relative difficulty of identifying sRNA-encoding loci by bioinformatic approaches. First, unlike protein-encoding genes, sRNAs do not encode open reading frames and thus cannot be readily identified based on their primary sequence alone. Second, unlike rRNAs and tRNAs, which are well conserved among diverse bacterial species, most known sRNAs are conserved only among closely related species. Since the vast majority of sRNAs physically confirmed to date have been identified in only a few species of γ-proteobacteria, prediction of sRNAs based solely on homology to known sRNAs has yielded relatively few loci outside of this class [4], [5].

Several bioinformatic approaches have been developed to identify sRNA-encoding genes in intergenic regions of bacterial genomes by searching for the co-localization of genetic features such as predicted Rho-independent transcription terminators, promoters and transcription factor binding sites (TFBSs), intergenic conservation among closely related species, and/or conserved secondary structure [3]. While these bioinformatic approaches have proven effective in identifying sRNAs in several species, they present significant computational challenges, requiring the positional relationships of thousands of individual genetic features to be ascertained for each genome-wide search. Several computational programs, including sRNAPredict2 and ISI [6], [7], have recently been developed to facilitate the efficient execution of these algorithms. However, searches using these programs involve highly fragmented protocols requiring execution of numerous interdependent programs. Moreover, some of the steps in these algorithms are computationally demanding. Prediction and annotation of sRNA-encoding genes in a single genome using currently available tools therefore requires significant user supervision and can take several hours to complete; conducting these searches in the hundreds of sequenced bacterial genomes would take many thousands of hours of supervised computation. Furthermore, previous algorithms designed to identify sRNA-encoding genes provide little functional annotation of predicted loci and thus yield little insight into the potential biological roles of candidate loci.

To enable high-throughput, kingdom-wide prediction and functional annotation of bacterial sRNA-encoding genes, we developed SIPHT (sRNA identification protocol using high-throughput technologies). SIPHT utilizes automatic workflow and distributive computing to enable a single user to conduct rapid kingdom-wide searches for sRNAs, a task that would not be practical without these computational approaches. SIPHT identifies candidate intergenic loci based on the co-localization of intergenic conservation and Rho-independent terminators and then annotates each of these loci for numerous features designed to provide information regarding the strength of its prediction and/or its potential biological functions. Using SIPHT, predictions and annotations of putative sRNA-encoding genes were conducted in 932 bacterial replicons yielding 45,599 candidates for previously unannotated intergenic loci. Annotations of these loci by SIPHT help to identify particularly strong candidates for novel intergenic transcripts and differentiate between putative sRNA-encoding genes and other types of intergenic loci such as conserved untranslated regions (UTRs) of mRNAs, intergenic repeat sequences, and cis-acting regulatory RNAs. Functions for thousands of the predicted loci, including several previously confirmed but uncharacterized small intergenic transcripts, are suggested by their homology to and/or shared synteny with genes encoding characterized sRNAs or cis-regulatory RNA elements, their association with putative TFBSs, and/or their pattern of conservation both within the same genome and among the genomes of other species. These kingdom-wide predictions and annotations, along with a web-interface allowing access to the SIPHT system are available at http://bio.cs.wisc.edu/sRNA. The SIPHT interface is accessible using the login name and password “SIPHT” and search results will be sent to the account SIPHTreviewergmail.com accessible using the password “reviewer”.

## Methods

### Genome sequence and annotation files and sRNA databases

Genome sequence and ORF sequence files (.fna and .ffn extensions, respectively) and annotation files (.gbk extensions) were obtained from the NCBI ftp database. All sequences and coordinates of confirmed *E. coli* sRNAs were obtained from the EcoCyc database [8] or from published results [9], [10] and of tested but unconfirmed *E. coli* loci from published results [11], [12]. Sequences and coordinates of physically tested sRNAs in *V. cholerae, P. aeruginosa, B. subtilis, L. innocua, P. marinus*, *S. typhimurium* and S. *aureus* were based on published results [7], [13]–[20] and in *S. meliloti* on both published results [21] and unpublished results (C. Valverde, J.L., J. Schlüter, J. Reinkensmeier, A. Becker and G. Parisi). A database combining the coordinates of these physically confirmed sRNAs with the coordinates of putative non-coding RNAs (ncRNAs) obtained from Rfam version 8.1 [4] and from the predictions of Weinberg *et al.* [22] was used to identify predicted loci corresponding to previously predicted and/or experimentally detected ncRNAs and to annotate for homology to and conserved synteny with ∼350 experimentally confirmed or putative sRNAs and cis-encoded regulatory RNAs.

### Identifying primary conservation

Intergenic regions (IGRs) in each replicon were compared using BLASTN 2.0 [9] (with E set to 5e-3 and B and V each set to 10,000) to a database including all IGRs in all other replicons except i) IGRs from the same strain ii) IGRs from different strains of the same species (strains sharing the same genus and species names) and iii) IGRs with an AT or GC content above 75%. The last class of IGRs was excluded from BLAST comparisons because these IGRs were found to produce high scoring alignments with IGRs in numerous unrelated AT or GC-rich species that are unlikely to correspond to real sequence conservation. Overlapping predicted loci conserved in multiple replicons were parsed into a single locus; the reported E value and score for each locus corresponds to the lowest E value and highest score of these overlapping candidates. In the annotation of candidate loci for inter- or intragenomic sequence conservation, BLAST comparisons were conducted not between the sequences of the putative RNA-encoding loci, which were often quite short, but between the entire IGR sequences containing these loci. The BLAST E threshold in these comparisons was set to 1e-3.

sRNA_Annotate searches the output of this IGR vs. IGR BLAST to identify sequence alignments that overlap the RNA-encoding genes in both the query and database IGR sequences. Two loci in different replicons are considered homologous if, for both loci, the region of overlap between the BLAST alignment and the locus is *i)* more than 50% of the entire length of the locus, *ii)* more than 80% of the entire length of the BLAST alignment, or *iii)* longer than 75 bp. Two loci in the same replicon are considered homologous if, for both loci, the region of overlap between the BLAST alignment and the loci is more than 20% of the entire length of the locus.

### Terminator identification

Three programs, RNAMotif, TransTerm, and FindTerm, were used to identify putative Rho-independent terminators. RNAMotif searches were conducted using RNAMotif v3.0.4 [23] with a motif descriptor provided by D.J. Ecker. TransTerm searches were conducted using TransTermHP v2.05 [24]. The FindTerm program was created by Gilgi Friedlander based on a heuristic algorithm developed by Ruth Hershberg [11].

### Identifying conserved secondary structure

IGRs containing candidate sRNA loci were compared by BLAST to a database of all IGRs. QRNA analyses were conducted using version 2.0.3d [25] with the window size (w) set to 150 and the slide size (x) set to 50. A putative sRNA is reported to correspond to a region of conserved secondary structure (denoted as ‘RNA’ in the ‘QRNA?’ column of the output file) or coding region (denoted as ‘COD’ in the ‘QRNA?’ column of the output file) if that sRNA overlaps any region predicted by QRNA to encode conserved secondary structure or conserved coding sequence, respectively, above a score of 5. If a candidate overlaps both a region predicted as RNA and a region predicted as COD, it is annotated as RNA/COD or COD/RNA, with the highest scoring prediction listed first.

### Search parameters for sRNA prediction

In all searches, the maximum overlap between a predicted locus and an annotated ORF was set at 65 and the maximum gap allowed between the 3′ end of a region of conservation and the 5′ end of a predicted Rho-independent terminator was 35 bp. The maximum E value, the minimum TransTerm confidence value, maximum RNAMotif and FindTerm scores, and minimum and maximum lengths of predicted loci were set to the following values: Search A- 1, 80%, -6, -7, 30, and 600; Search B- 1e-5, 86%, -7.5, -10, 35, and 550; Search C- 1e-15, 87%, -9, -10, 50, and 500; Search D- 1e-25, 90%, -10, -11, 60, and 450. Loci predicted in IGRs longer than 1 kbp were excluded as this was found to significantly increase specificity. The reported 5′ end of the predicted locus is based on the 5′ border of the shortest HSP associated with that locus; the 3′ end of the predicted locus is based on the predicted 3′ end of its associated terminator. Thus, the reported lengths of putative loci may differ significantly from their actual size. Loci predicted overlapping but antisense to other loci were discarded if 1) they were associated with a terminator identified by only one terminator prediction program and 2) their antisense loci was associated with a terminator predicted by >1 program.

### Identifying TFBSs

BioProspector [9], [26] was used to search for conserved motifs upstream of genes known to be regulated by particular transcription factors, to generate TFBS consensus sequences. For TFBS consensus motifs of variable length, separate matrices were constructed for each motif length. Lists of genes regulated by *B. subtilis* SigA, SigK, and SigG were obtained from DBTBS [27]. Lists of genes regulated by LexA and Fur were based on their experimentally determined regulons in both gram-positive and gram-negative species (for Fur [28]–[31]; for LexA [32]–[34]). The list of genes regulated by σ^54^ in diverse species was obtained from Barrios *et al.* [35]. The list of genes regulated by σ^E^ and σ^70^ in *E. coli* were obtained from Rhodius *et al.* [36] and from RegulonDB [37], respectively. Searches for putative TFBSs corresponding to each consensus matrix were conducted using Patser v3e.1 [38]. For Patser searches, the a *priori* nucleotide probabilities used to convert the alignment matrix to a weight matrix were set to 0.25 for all 4 nucleotides. Annotations for Fur, LexA, and σ^54^ binding sites were conducted for all strains; for σ^70^ and SigA, gram-negative and gram-positive strains, respectively; for SigK and SigG, sporulating strains; for σ^E^, strains of *Enterobacteriaceae*.

### Determining conserved synteny

Nucleotide sequences of the two ORFs closest to the 5′ end and the two ORFs closest to the 3′ end of candidate and previously annotated or confirmed loci were compared by BLAST (E<1e-3). In annotating predicted loci as sharing conserved synteny with known ncRNA-encoding loci, homology and conserved orientation relative to EITHER the nearest upstream OR nearest downstream gene of the previously identified locus was regarded as sufficient. Loci sharing homologous genomic context and orientation with only one flanking ORF are denoted in the text as having ‘conserved 3′ synteny’ or ‘conserved 5′ synteny’, respectively; a locus is said to have ‘conserved synteny’ with another locus ONLY if at least one of the two nearest upstream genes AND at least one of the nearest downstream genes are homologous AND their orientation relative to these homologous genes on both sides is conserved.

## Results and Discussion

### A summary of SIPHT, a high-throughput program for predicting and annotating bacterial sRNA-encoding genes

**Figure 1:**
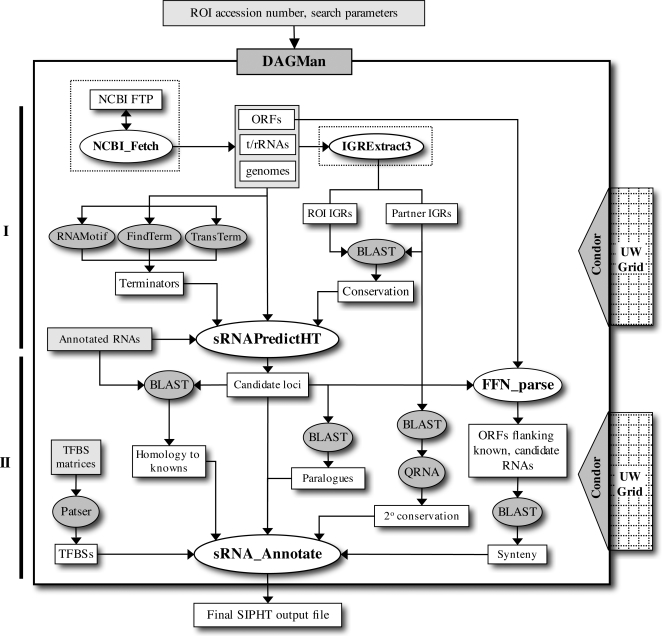
Schematic of SIPHT. The two main stages of the SIPHT protocol are shown on the left. The two sets of non-interdependent programs in the workflow that are executed in parallel are denoted by shaded ovals. The steps in the workflow surrounded by dotted lines are not executed in every search but rather periodically to update local databases.

A schematic of the SIPHT protocol is shown in Figure 1. Three programs, RNAMotif, TransTerm, and FindTerm, are used to predict Rho-independent terminators. Conserved intergenic sequences are identified by comparing the IGRs of the replicon of interest (ROI) to all appropriate IGRs from other replicons using BLAST (see Methods). sRNAPredictHT then identifies candidate sRNA-encoding genes by searching IGRs for putative terminators located within or directly downstream of conserved sequences and annotates these candidates for the following features: the coordinate position and strand orientation of each predicted sRNA-encoding gene; the distances between the candidate gene and the genes flanking its IGR as well as the name and orientation of these genes; the BLAST E value and score of its associated conserved sequences; the number and name(s) of the replicons in which it is conserved; which program(s) predicted its associated terminator; and whether it corresponds to a previously annotated regulatory RNA. Following candidate prediction and annotation by sRNAPredictHT, several other programs including BLAST, QRNA, Patser, and FFN_parse are used to annotate the candidate genes for a number of features including i) homology to previously identified regulatory RNAs ii) homology to other candidates in the same replicon iii) association with putative transcription factor binding sites (TFBSs) iv) conserved secondary structure and v) conserved synteny with previously identified regulatory RNAs. The output of the SIPHT search is a tab-delimited file that can be opened in Excel and that allows candidate loci to be sorted by any of the features described above.

As shown in Figure 1, the entire SIPHT workflow was unified and automated using DAGMan (Directed Acyclic Graph Manager), a scheduler that coordinates the execution of a set of programs that are inter-dependent due to input/output constraints [39]. Management of the SIPHT workflow by DAGMan allows the user to conduct an entire SIPHT search in one replicon or in over one thousand replicons by a single invocation of a command line. This command line is used to create a configuration file that is accessed by various programs in the workflow. While DAGMan allows SIPHT to be run as a unified, “launch and leave” process, several of the steps in the SIPHT protocol are computationally intensive and can each take several hours to complete for a search of a single replicon. Thus a single SIPHT search of all 932 bacterial replicons would take over two months to complete if executed on a single CPU. To increase the throughput of SIPHT searches, distributed computing was incorporated into the SIPHT protocol using Condor, a distributed batch system that manages the allocation and execution of computational tasks to distributed systems like computational grids [39]. By utilizing automated workflow management and harnessing the collective computing power of over 1500 computing cores in the Grid Laboratory of Wisconsin (GLOW), SIPHT is able to complete a search in a typical genome in 1–2 hours and in all 932 sequenced bacterial replicons in the NCBI database in less than 12 hours without any user intervention. Each kingdom-wide analysis consumes over 1600 computing hours (more than 60 computing days), incorporates tens of thousands of individual data files, and requires 11 different scripts to be executed a total of over 12,000 times and thus would not be feasible without the high-throughput capabilities employed by SIPHT.

### Calibrating the SIPHT search parameters

The SIPHT protocol incorporates numerous adjustable search parameters, including the maximum E value of BLAST alignments, the minimum score or confidence thresholds for terminator predictions, and the minimum and maximum lengths of predicted loci. To determine appropriate search parameters for our kingdom-wide searches, we conducted 4 SIPHT searches in 8 diverse species, increasing the stringency of each search by coordinately adjusting the parameters described above (Searches A–D; see Methods). The 8 species chosen for these analyses were ones in which several intergenic loci had been subjected to experimental validation and/or had been previously annotated based on sequence homology to experimentally confirmed intergenic transcripts. In several of these species, a significant number of candidate loci that had been subjected to experimental testing had not been confirmed, suggesting they corresponded to false predictions (referred to throughout as false predictions).

**Figure 2:**
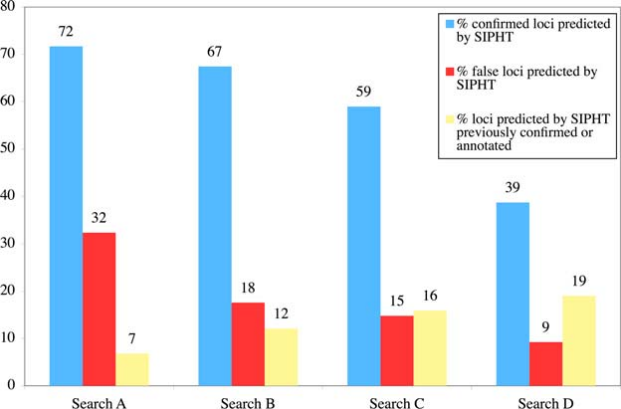
Influence of variations in search parameters on SIPHT predictions. The search parameters in searches A–D become increasingly more stringent and are listed in the methods section.

As shown in Figure 2, the lowest stringency search (Search A) yielded over 70% of 212 confirmed transcripts but also 33% of 108 false predictions, suggesting that searches employing these parameters have a high sensitivity but low specificity for real sRNA-encoding loci. The most stringent search conducted, Search D, yielded a marked reduction in sensitivity for confirmed loci (Figure 2), especially *in V. cholerae* and *P. aeruginosa*, where the proportion of confirmed transcripts identified dropped from 60% to 10% and from 79% to 14%, respectively. These observations suggest that the parameters used in Search A and Search D were not stringent enough and too stringent, respectively. In contrast, the search parameters used for Searches B and C yielded only modest reductions in sensitivity compared with Search A but significantly improved specificity (Figure 2). The parameters used in Search C were employed in our kingdom-wide searches because these parameters yielded higher specificity than those used in Search B. The proportion of confirmed loci identified in Search C varies significantly among the 8 species included in this search (Table 1). In general the sensitivity of the SIPHT predictions were markedly higher in proteobacteria than in other species. Of the known sRNAs not predicted, most were missed because their associated terminator was not identified. This was particularly true for the sRNAs missed in Gm^+^ species.

**Table 1:** Comparison between loci predicted by SIPHT and those previously subjected to experimental validation in 8 diverse species.

	Total # experimentally confirmed^a^	% confirmed predicted by SIPHT	Total # tested but not detected	% unconfirmed predicted by SIPHT
*E. coli K12*	72	**51.4**	83	**13.3**
*B. subtilis*	4	**50.0**	0	**-**
*V. cholerae*	14	**78.6**	4	**0.0**
*P._aeruginosa*	20	**60.0**	11	**9.1**
*S. aureus N315*	8	**12.5**	0	**-**
*S._meliloti*	15	**66.7**	0	**-**
*S. typhimurium*	66	**72.7**	10	**40.0**
*L. monocytogenes*	13	**30.8**	0	**-**
**Total**	212	**59.0**	108	**14.8**

a These numbers include sRNAs detected by Northern analysis and do not include homologues of highly conserved “housekeeping” sRNAs such as tmRNA, RnpB, 4.5S and 6S RNA, or the *E. coli* sense/antisense Sok and Rdl sRNAs. These numbers correspond to searches conducted using Search C parameters.

### Over 45,000 candidate loci were identified in the kingdom-wide SIPHT searches

**Figure 3:**
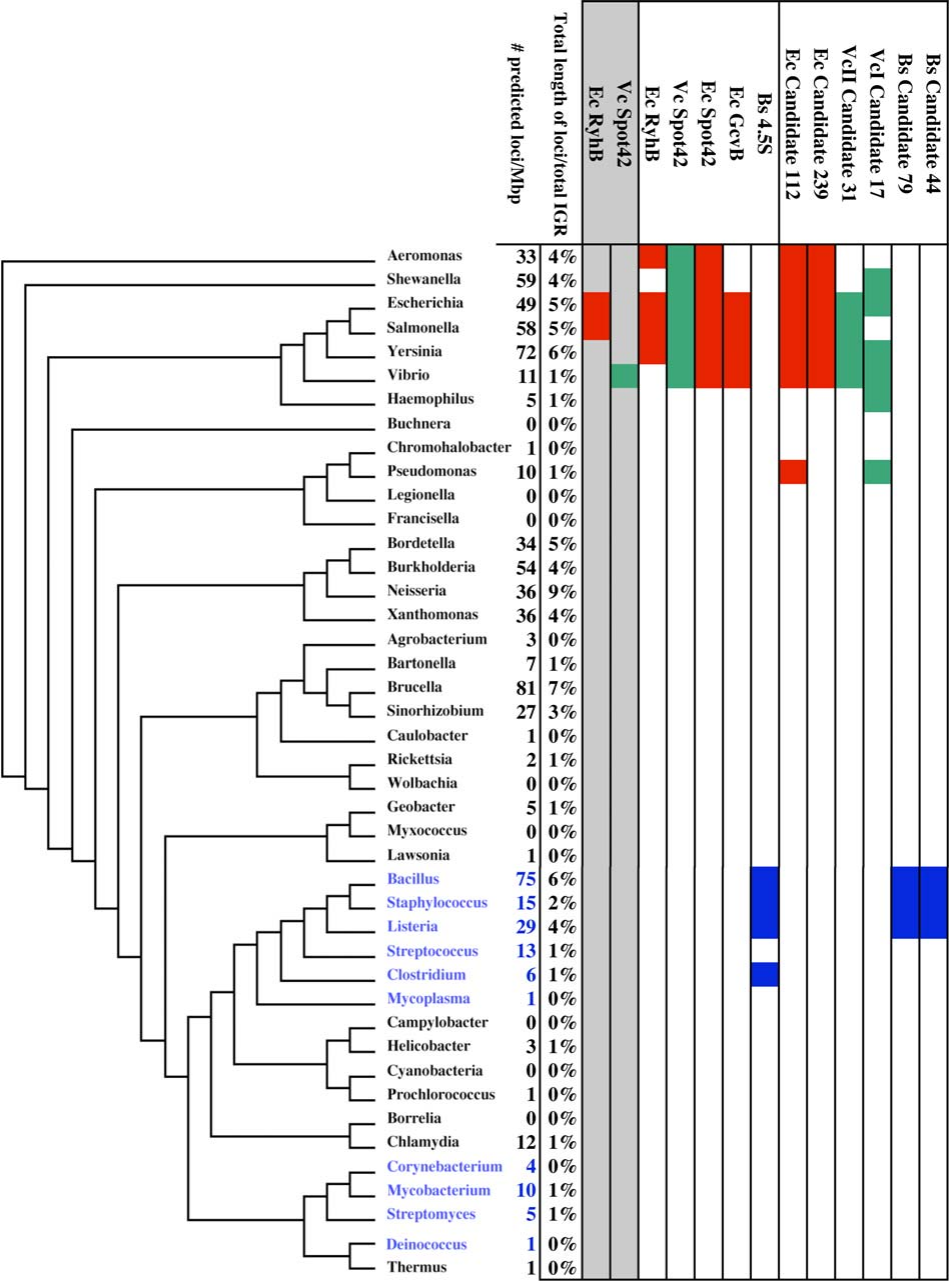
Phylogram showing variations in the densities of predicted loci and in the conservation of known and candidate loci among diverse bacterial genera. The phylogram is based on the 16S RNA sequences of a representative species in each genera. Gm+ and Gm− genera are colored blue and black, respectively. BLAST analyses were performed for known and candidate loci from *E. coli* (Ec), *V. cholerae* (VcI for chromosome I, VcII for chromosome II), and *B. subtilis* (Bs), which are colored red, green, and blue, respectively. Filled boxes denoted that the locus was predicted based on intergenic conservation in the indicated genera. Columns shaded gray and unshaded columns show results with BLAST E set to 1 e-15 and 1e-3, respectively.

The SIPHT output files for each of the 932 bacterial replicons included in our search, instructions for interpreting the data in these files, and a web-accessible interface to SIPHT are available at http://bio.cs.wisc.edu/sRNA. The SIPHT kingdom-wide searches yielded a total of 47,273 loci, including 45,599 loci not corresponding to previously tested sRNAs or included in the Rfam database (v. 8.1) (referred to throughout as candidate loci). This database contains putative homologues of previously confirmed intergenic loci identified using a BLAST-based heuristic [4]. These candidate loci were identified in the chromosomes of 461 of the 524 strains included in our search and in 260 of 364 plasmids. Many of the genera in which no loci were predicted are obligate intracellular symbionts such as *Mycoplasma* sp. and *Buchnera* sp.; it is unclear whether the lack of predicted loci in these species reflects an absence of RNA-mediated regulation or limitations of our predictive algorithm in identifying regulatory RNA loci in these organisms with reduced genomes. An average of 25 loci per megabasepair of chromosomal sequence were predicted in the SIPHT searches; however, the density of chromosomal loci predicted among different species varied significantly, in some cases even among species in the same genera (Figure 3 and Table S1). Phylogenetic analysis of our results revealed that species with a high density of predicted loci tend to be clustered among closely related genera of certain phyla such as γ- and β-proteobacteria and firmicutes. In certain cases, the variation in locus density can be accounted for by differences in the number of predicted intergenic terminators and differences in the total amount of intergenic conservation. This was particularly true for variations among species in the same genera. For example, the nearly 50-fold difference in the number of loci predicted among Bacillus species was almost entirely attributable to differences in the amount of IGR conservation (Table S1). However, much of this variation persisted even after normalization for IGR conservation and terminator density. These findings suggest that RNA-mediated regulation may be more prevalent in certain genera than in others.

### Estimating the accuracy of the SIPHT predictions

While the predictive approach employed in our kingdom-wide searches have proven effective in identifying sRNA-encoding genes in diverse species, they have often yielded a substantial proportion of false predictions [5], [7], [13], [18]. Due to the scope of our analyses, employing experimental methods to determine the rate of false predictions in all or even a representative sample of species is unfeasible. To assess the accuracy of the SIPHT predictions, we estimated the maximum false positive rate for the 8 species described above by determining the proportion of loci predicted in our searches that correspond to experimentally detected transcripts or putative intergenic loci annotated in Rfam (Table 2). In most of these species, this proportion ranged between 11% and 25%. If one assumes all regulatory RNAs expressed by these species have been previously detected and/or annotated in Rfam, these findings suggest that the maximal false positive rate among these species is between 75% and 89%. However, it is unlikely that the database of previously detected regulatory RNAs and their homologues represents a comprehensive catalogue of all non-coding transcripts. The experimental screens for *E. coli* sRNAs have either been limited to highly abundant transcripts or have specifically targeted transcripts that interact with the RNA chaperone Hfq [40]–[43]. In addition, hundreds of intergenic *E. coli* loci predicted in computational screens, many of them identified by two or more distinct bioinformatic algorithms, have never been subjected to experimental validation [5], [10], [44]. Thus, even in *E. coli*, the species in which the most extensive computational and experimental screens for sRNAs have been conducted to date, it is likely that only a subset of regulatory RNAs have been identified. Indeed, two recent studies have estimated that *E. coli* expresses a total of 150–200 sRNAs [10], [44].

**Table 2:** Proportion of loci predicted by SIPHT corresponding to previously detected or annotated RNA-encoding loci.

	HR included^a^		HR excluded^a^	
	# loci predicted by SIPHT	% previously detected or annotated^b^	# loci predicted by SIPHT	% previously detected or annotated^b^
*E. coli K12*	401	**11.0**	308	**13.8**
*B. subtilis*	155	**25.2**	136	**24.3**
*V. cholerae*	78	**43.6**	42	**57.1**
*P. aeruginosa*	32	**51.6**	32	**51.6**
*S. aureus N315*	61	**23.0**	47	**29.8**
*S. meliloti*	218	**6.4**	186	**7.5**
*S. typhimurium*	410	**12.3**	355	**13.9**
*L. monocytogenes*	124	**18.5**	122	**18.9**
**Total**	1479	**15.9**	1228	**17.6**

a HR loci share homology with more than 4 other loci predicted in different IGRs but in the same replicon.

b % of the total number of loci previously experimentally confirmed or annotated in the Rfam database.

As shown in Table 2, there is a considerable range in the ratios of confirmed to total predicted loci in different species. This ratio was greater than 50% for *V. cholerae* and *P. aeruginosa* and lower than 10% for *S. meliloti*. The high ratio in *V. cholerae* reflects the inclusion in this analysis of many novel small transcripts we have recently identified in this organism using an unbiased high-throughput sequencing approach for small RNA discovery (J. Liu, J.L, M.K.W., and A. Camilli, unpublished data). Similarly, the relatively low ratio of candidate to previously identified loci in *S. meliloti* likely reflects the lack of comprehensive screens for sRNAs conducted in this organism as well as the relatively limited homology between *S. meliloti* and the species in which the vast majority of regulatory RNAs have been identified. It is also possible that the accuracy of SIPHT predictions varies among diverse species, perhaps due to species-dependent differences in the accuracy of terminator predictions or to differences in the availability of appropriate BLAST partners for identifying intergenic sequence conservation. As shown in Figure 2, the minimum accuracy of SIPHT predictions can be improved by increasing the stringency of the search parameters; however, as discussed above, this increased accuracy will often result in reduced sensitivity.

Not all SIPHT predictions that correspond to bona fide intergenic transcripts will correspond to sRNAs. Some predictions may correspond to conserved UTRs of mRNAs or conserved intergenic repeat elements; others may correspond to cis-encoded regulatory RNAs such as riboswitches. Riboswitches are regulatory elements encoded in the untranslated regions (UTRs) of mRNAs that typically modulate gene expression by switching between termination and anti-termination conformations in response to interactions with specific metabolites or, in the case of T-boxes, tRNAs [45]. As described below, SIPHT annotations of the predicted loci can be used to identify particularly strong candidates for sRNA-encoding genes, to differentiate between putative sRNA-encoding genes and other types of intergenic loci, and to provide insights into the potential biological roles and/or evolution of candidate regulatory RNA-encoding loci. Thus, in addition to increasing the stringency of the search parameters, filtering candidate loci using these annotations can significantly increase the accuracy of the SIPHT predictions (see below).

### Identification of strong candidates for regulatory RNA-encoding loci

As shown in Table 1, our searches yielded 16 presumed false predictions in several species. We reasoned that these 16 loci were likely identified by SIPHT based on false terminator predictions and/or on primary sequence homology that does not correspond to conserved RNA sequences or structures. Indeed, we found that if we limited our predictions to loci that are associated with terminators predicted by all three programs and predicted by QRNA [12] to encode conserved secondary structure (‘ZQ’ loci), 23 of the 125 (18%) experimentally confirmed loci but none of the 16 unconfirmed loci identified in our initial searches were predicted. Similarly, 28 (18%) of 152 confirmed loci but none of the 35 unconfirmed loci predicted in Search A were ZQ loci. Moreover, among all loci predicted in our kingdom-wide searches, the proportion of ZQ loci was higher among previously annotated or confirmed intergenic loci (23%) compared to candidate loci (14%) (Table S1). These finding suggest that limiting SIPHT predictions to ZQ loci, while markedly decreasing their sensitivity, also significantly increases their specificity. Thus, the 6,561 ZQ candidate loci predicted in our search represent particularly strong candidates for real small intergenic transcripts.

### Identifying loci corresponding to UTRs or intergenic repeat sequences

In previous studies, numerous loci predicted to encode intergenic sRNAs were ultimately shown to be UTRs of mRNAs by Northern analysis. To help identify predicted loci that may correspond to conserved UTRs rather than sRNA-encoding genes, SIPHT annotates all candidates overlapping a region up to 30 bp upstream of an ORF and/or associated with a terminator within 80 bps of its upstream ORF as potential 5′ and/or 3′ UTRs, respectively. A total of 8,028 candidate loci predicted by SIPHT were annotated as potential UTRs (Table S1), including 4 (25%) of the 16 unconfirmed loci predicted and 17 (14%) of the 125 confirmed loci identified. While 3 of the previously confirmed transcripts annotated as UTR loci (*E. coli sgrS* and *V. cholerae qrr1* and *spot42*) have been shown to function as trans-acting regulatory transcripts, previous reports have suggested that 4 others (*E. coli* and *S. typhimurium sroC* and *sraF*) may correspond to cis-regulatory RNAs rather than to sRNAs [43], [46].

SIPHT annotations also revealed that nearly 18% of the candidate loci share homology with more than 4 other loci predicted in different IGRs but in the same replicon (HR loci) (Table S1). Many of these loci correspond to previously described intergenic repeat sequences in *Burkholderia* sp. [47] and to enterobacterial repetitive intergenic consensus sequences (ERICS) in *E. coli*, *Yersinia* sp., and *Vibrio* sp. [48]–[50]. Previous studies have suggested that ERICS function as cis-acting RNA regulatory elements [48], [50]. Of the 226 HR loci previously annotated in Rfam, nearly all correspond to confirmed or putative riboswitches. Only 3 of the 125 confirmed transcripts identified in our searches were annotated as HR loci, and the functions of these small transcripts have yet to be determined [20], [40]. Since numerous replicons encode a significant number of HR loci (Table S1), filtering out HR loci can significantly decrease the number of candidate loci in numerous species. For example, as shown in Table 2, excluding HR loci in *E. coli* leads to a 26% reduction in the number of candidate loci; in *V. cholerae*, applying this filter leads to a 59% decrease in the number of candidate loci. Interestingly, most of the HR loci in *V. cholerae* were concentrated within the superintegron of chromosome II. The exclusion of HR loci increased the proportion of previously detected or annotated loci predicted in *E. coli*, *S. aureus*, and *V. cholerae*, suggesting this filter increases the specificity of SIPHT predictions for regulatory RNA-encoding genes in some species (Table 2). However, excluding HR loci did not increase the specificity of SIPHT predictions in all species (Table 2). Since the number of HR loci varies significantly among different species (Table S1), applying this filter to the SIPHT output can only be effective for those replicons in which a significant number of HR candidate loci were predicted.

### Identifying loci corresponding to putative riboswitches 

In addition to identifying most known sRNAs, SIPHT also identified 769 (31%) of 2447 riboswitches previously annotated in Rfam. The vast majority of riboswitches reported to date have been identified in 5′ UTRs in Gm^+^ species and most regulate the expression of biosynthetic enzymes or metabolite transporters. In *B. subtilis*, an organism in which riboswitches have been extensively studied [51], SIPHT identified 29 (58%) of the 50 riboswitches annotated in the Rfam database, including 27 (74%) of the 34 known or putative T-box, SAM, and TPP riboswitches.

Recent studies suggest that riboswitches regulate the expression of many more groups of genes in more diverse bacterial species than previously thought [22], [46]. In examining predicted loci corresponding to previously annotated cis-encoded regulatory RNAs, we found that the vast majority are *i*) encoded on the same strand as their 3′ ORF *ii*) predicted by QRNA to have conserved secondary structure *iii*) annotated as having at least one other homologue in the same replicon and *iv*) are found less than 100 bps upstream of the start codon of their associated gene. Thus, to help identify candidate loci corresponding to putative riboswitches, we modified the SIPHT protocol to annotate predicted loci as potential riboswitches (RS loci) based on their association with these 4 features and repeated the kingdom-wide searches. A total of 1,978 candidate RS loci were predicted in our searches. Additionally, 439 RS loci corresponding to previously annotated RNAs were identified by SIPHT, of which 419 (95%) correspond to confirmed or putative riboswitches. These included 22 (76%) of the 29 confirmed or putative *B. subtilis* riboswitches predicted. Importantly, only 1 of the 125 previously confirmed loci identified in our search (the *S. typhimurium omrA* homologue) were annotated as RS loci. Taken together, our findings suggest that RS annotations can be reliably used to help distinguish between loci corresponding to sRNAs and those corresponding to riboswitches.

In summary, a total of 29,780 candidate loci predicted in our searches were not annotated as RS, HR, or UTR loci, suggesting that they are more likely to correspond to sRNA-encoding genes than to other types of intergenic loci. Of these 29,780 loci, 3,919 are ZQ loci and thus represent particularly strong candidates for novel sRNA-encoding genes.

### Adjusting BLAST stringency yields more sensitive annotation of locus conservation and reveals candidate loci with unusual patterns of sequence conservation

Each locus predicted by SIPHT is annotated for the number and names of all replicons in which it was found to be conserved in the initial SIPHT BLAST search. Loci corresponding to housekeeping RNAs such as tmRNA, RnpB, and 4.5S RNA were among the most highly conserved, along with homologues of *E. coli* Spot42 [52], RyhB [53] and GcvB [54]. However, with the BLAST parameters used for the kingdom-wide search, the extent of conservation of several previously characterized sRNAs as annotated by SIPHT was more limited than expected based on previous reports. For example, *E. coli* RyhB, which has been shown to be conserved in numerous genera of *Enterobacteriaceae*, was not conserved in *Y. pestis* and *V. cholerae* Spot42, which was identified based on its homology to *E. coli* Spot42 was only conserved among other *Vibrio* species (Figure 3). We reasoned that this was likely due to the relatively high BLAST stringency used in our kingdom-wide searches. Indeed, when SIPHT searches were repeated with the BLAST E value set to 1e-3, the patterns of conservation of these loci were consistent with previously published results (Figure 3). These findings suggest that while a high BLAST stringency yields more accurate predictions of sRNA-encoding loci, lowered BLAST stringency may be useful in more sensitively identifying primary sequence conservation of these loci in less closely related species.

Based on the results above, we repeated the SIPHT searches with the BLAST E value set to 1e-3. Even at this reduced stringency, most confirmed sRNAs and candidate loci identified were conserved only among closely related species. However, as shown in Figure 3, SIPHT identified several candidate loci that, like RyhB, Spot42 and GcvB [54], are conserved among more diverse genera. None of the candidate loci shown in Figure 3 share significant homology with previously characterized RNAs, suggesting they may represent new classes of well-conserved sRNAs.

### Annotation of primary sequence homology to and conserved synteny with known regulatory RNAs gives insights into the potential function and evolution of thousands of candidate loci

A total of 5,359 predicted loci were found to share significant homology with previously predicted or confirmed sRNAs or cis-encoded regulatory elements, including 3,962 loci not previously tested or annotated in Rfam (Table S2). With the exception of homologues of well-conserved RNAs such as tmRNA, RnpB, and Spot42, homologues of known sRNAs were generally found only among strains in the same genera or, in the case of *E. coli,* in the same family. Thus, consistent with previous findings [4], [5], we found that using primary sequence conservation to known regulatory RNAs has limited efficacy in deciphering the function of candidate loci among most of the diverse species included in our analyses.

**Figure 4:**
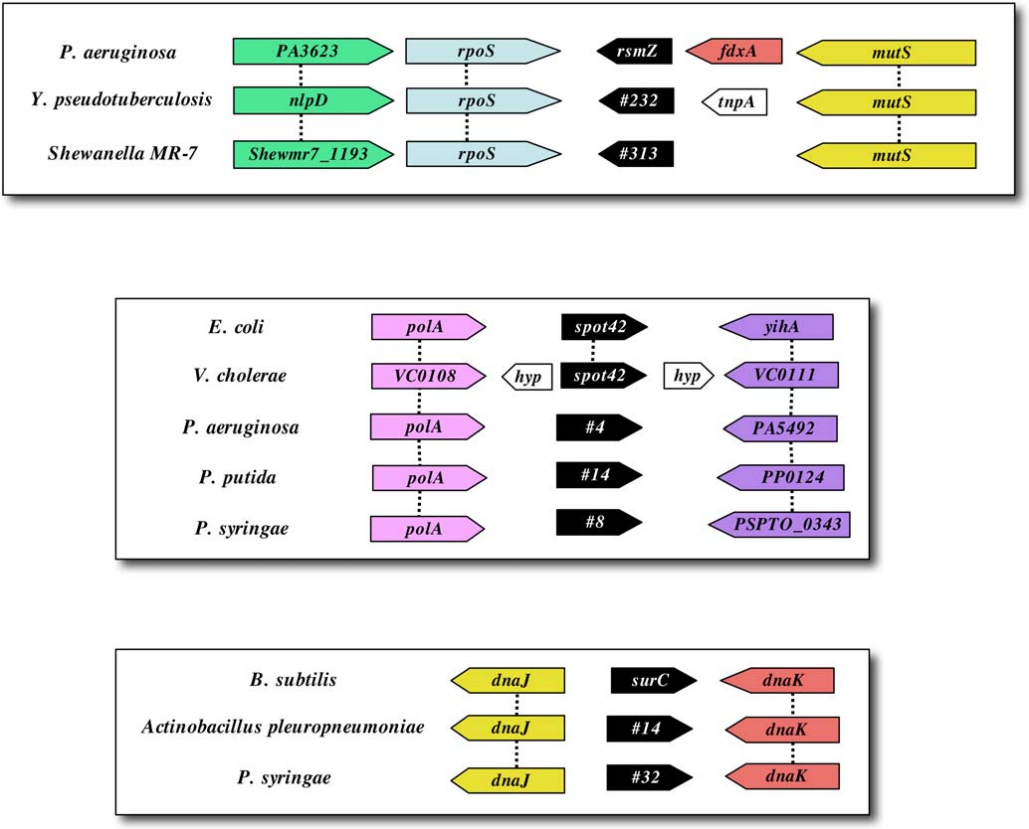
Examples of conserved synteny between candidate loci and previously identified sRNAs. Predicted loci are colored black; the previously annotated name or candidate number for each locus is indicated. ORF names are based on NCBI annotations and dashed lines connect homologous ORFs (BLAST E<1e-3). Additional candidate loci with conserved synteny are shown in Table S3.

In most cases, homologous sRNAs encoded by closely related species share conserved synteny (i.e. are flanked by homologous ORFs and encoded in the same orientation relative to these ORFs (see Methods)). Interestingly, we found 3,562 loci that share conserved synteny with but no significant primary sequence homology with previously annotated or confirmed RNA-encoding loci (Table S3). These include candidate loci in diverse species encoded in the same genomic context as the *E. coli* sRNAs Spot42, SgrS, SraA, SraG, and tff, *P. aeruginosa* RsmZ, and *B. subtilis* SurC (Figure 4 and Table S3). Several previously confirmed loci were also found to share conserved synteny but not sequence homology with other known loci. For example, SraG was found to share conserved synteny with the sRNA RliD recently confirmed in *Listeria monocytogenes* [55], and the *P. aeruginosa* sRNA P15 [7] was found to share conserved synteny with a TPP riboswitch in *L. monocytogenes*, suggesting it may correspond to a riboswitch rather than a trans-encoded sRNA. It seems likely that non-homologous loci with conserved synteny arose from a common ancestral gene but have since diverged at the level of their primary sequence. Previous studies have shown that non-homologous sRNAs encoded by different species can still perform analogous regulatory roles and even target homologous mRNAs [15], [53], [56]; thus it is possible that syntenous sRNA-encoding loci still share conserved regulatory functions even in the absence of primary sequence homology. Conserved genomic context may therefore be useful *in lieu* of primary sequence homology in annotating putative candidate loci for their potential biological functions. This is particularly true for the 1,138 loci that share conserved 3′ synteny but no significant homology with previously identified cis-acting regulatory RNAs (Table S3), since the function of these elements is dependent on their genomic context.

### Identification of predicted loci associated with putative transcription factor binding sites

SIPHT identified 740 previously confirmed or annotated loci associated with putative TFBSs. Twenty-one of 22 alternative sigma factor or transcription factor binding sites associated with known sRNAs were identified in our search. Moreover, of the 22 putative σ^70^-dependent or SigA-dependent promoters identified upstream of known sRNAs, the great majority were within 10 bp of the transcriptional start site as experimentally determined or as predicted based on the observed length of the sRNA and the position of its predicted terminator. In addition to identifying known sRNA-associated TFBSs, SIPHT identified several previously unannotated putative TFBSs upstream of confirmed sRNA-encoding genes that may provide clues into the function of these uncharacterized sRNAs. For example, an RpoE-dependent promoter was found associated with the *E. coli* sRNA RygC [57] and in the same relative position upstream of putative homologues of RygC in several other species. Moreover, putative σ^54^ binding sites were predicted upstream of P11 and P30 [7] in *Pseudomonas* sp. and of sraJ/ryiA [11], [57] in several strains of *Enterobacteriaceae*.

Most of the candidate loci predicted in our searches do not share significant homology or conserved synteny with previously characterized sRNAs or riboswitches. To begin to elucidate the potential roles of these candidate loci, we analyzed whether they are associated with putative binding sites for σ^54^, LexA, and Fur. These transcription factors are well-conserved across both Gm− and Gm+ species and their regulatory roles have been determined in diverse bacteria. Moreover, all three have been shown to regulate the expression of previously characterized sRNAs.

σ^54^ is an alternative sigma factor that regulates numerous functions including motility and nitrogen fixation. σ^54^ has recently been shown to control the expression of several *V. cholerae* sRNAs involved in quorum sensing [58]. Putative σ^54^ binding sites were associated with 121 candidate loci (Table S4), including several previously unannotated homologues of Qrr loci [58] in *Vibrio* sp.

LexA is a global regulator of the SOS response. In *E. coli*, LexA has been shown to inhibit expression of the sRNA IstR2 [59]. A total of 113 candidate loci associated with putative LexA boxes were identified in both gram-negative and gram-positive species (Table S4), including several candidate loci predicted in several species of *Enterobacteriaceae* that share significant homology with IstR2. Presumably, many of the remaining candidates correspond to LexA-regulated sRNA loci that are involved in the SOS response.

Fur modulates expression of genes involved in iron homeostasis. In several gram-negative species, Fur mediates the upregulation of numerous genes through its repression of sRNAs that negatively modulate mRNA stability [15], [30], [53], [56], [60]. Our searches yielded a total of 325 loci associated with putative Fur binding sites (Table S4). Seventy-four of these showed significant homology to previously characterized Fur-regulated sRNAs (FRSs) in *E. coli*, *V. cholerae*, *P. aeruginosa*, and *N. meningitidis* [15], [30], [53], [56], [60]. These included loci corresponding to all previously known FRSs (RyhB, NrrF, and the PrrF loci), nearly all homologues of these FRSs in the Rfam database, and 36 previously unannotated candidate loci. For all previously characterized FRSs, the location of the putative Fur box relative to the predicted terminator was consistent with their experimentally determined lengths. Strikingly, for 73 of the 74 loci sharing homology with known FRSs, including all loci corresponding to characterized FRSs and previously annotated FRS homologues, the putative Fur binding site overlaps a putative σ^70^ promoter with the predicted 3′ end of the Fur box located exactly 1 nucleotide downstream of the predicted 3′ end of the putative σ^70^ promoter. These findings suggest a highly conserved organization of FRS operators. Further analysis revealed 26 additional loci that are associated with the canonical FRS operator described above but that do not share significant homology with known FRSs. These include candidate loci in *Bacillus* sp. that represent the first putative FRSs identified in Gm^+^ species. They also include the first putative FRSs identified in *Haemophilus*, *Actinobacillus*, and *Lactobacillus* species.

### Conclusions

By taking advantage of automated workflow management tools and distributed computing capabilities, SIPHT is the first computational tool that enables kingdom-wide prediction and annotation of intergenic RNA-encoding loci. The prediction of candidate intergenic loci by SIPHT in nearly 800 replicons in the NCBI database helps to fill significant gaps in the current annotations of bacterial genomes. Moreover, annotation of these loci for several features offers clues into the potential functions of many of these predicted loci and provides insights into the evolution of ncRNAs among diverse bacterial species. SIPHT also represents the first web-accessible tool by which researchers interested in studying RNA-mediated regulation can flexibly conduct searches for intergenic RNA-encoding loci in their species of interest. The SIPHT web-interface allows 19 different search parameters to be modified, including several affecting the stringencies of terminator predictions and BLAST, and thus will be particularly valuable to those researchers who want to adjust the sensitivity or specificity of their searches. By greatly enhancing the throughput and accessibility of ncRNA prediction and enabling more comprehensive annotations of both known and putative regulatory RNA-encoding loci, SIPHT provides a unique and important resource for the many groups studying RNA-mediated regulation in diverse bacterial species.

While examples of RNA-mediated regulation have been found in all branches of life, we are only now beginning to recognize the importance and ubiquity of this regulatory paradigm. The SIPHT-generated predictions discussed here generate many questions that warrant future experimental investigation. First, what is the number and variation of ncRNAs per genome? We identified candidate intergenic loci in the vast majority of bacterial replicons. Most genomes of 3–4 Mbp were predicted to contain ∼80–300 candidate ncRNAs, corresponding to about 3%–10% of the total number of predicted ORFs. Our findings also suggest that the density of ncRNAs varies among different species, but how significantly these apparent differences are determined by inter-species variations in sequence homology and recognizable Rho-independent terminators is unclear. Second, do syntenous but non-homologous sRNAs (e.g. the loci shown in Figure 4 and listed in Table S3) target homologous mRNAs and/or regulate similar functions? If so, characterizing the interactions between these non-conserved but functionally homologous sRNAs and their cognate target transcripts will give insights into sRNA-mRNA co-evolution and help elucidate the sequence and structural constraints governing RNA-mediated regulation. Finally, how widespread is riboswitch-mediated regulation and how diverse are the processes that these cis-acting elements control? Riboswitches have traditionally been thought to modulate relatively few pathways, mainly those involved in biosynthesis and transport, and to be significantly more common among Gm+ species. However, consistent with recent findings by Weinberg *et al.* [22], our analyses suggest that riboswitches may govern more diverse processes and be more widespread among Gm− organisms than previously believed.

While many thousands of novel candidate ncRNAs were identified by SIPHT, many *bona fide* sRNA-encoding loci were likely missed in these searches because they overlap real or misannotated ORFs, are not well conserved, and/or are not associated with predictable transcription terminators. Presumably, these loci may be amenable to prediction by algorithms that do not rely on one or more of these parameters. The modular design of SIPHT will allow it to be easily modified to incorporate new programs and novel predictive algorithms designed to discover more elusive classes of sRNAs. This modularity will also allow new programs and/or improved versions of existing programs to be added to SIPHT to improve the reliability of its predictions and annotations. The speed and ease with which searches can be executed using SIPHT will enable kingdom-wide predictions and annotations to be updated frequently as new data become available. Thus, SIPHT predictions and annotations will become increasingly effective as more genomic sequences become available, as more regulatory RNAs are confirmed and characterized, and as new TFBS consensus sequences are determined. In addition to facilitating the study of ncRNAs, SIPHT also serves as a reference framework for the development of a new generation of bioinformatic tools. As genome sequence databases continue to expand at rapidly increasing rates, computational approaches such as those employed by SIPHT that effectively integrate and efficiently execute a variety of novel and pre-existing bioinformatic tools will become indispensable in our attempts to decipher the complex regulatory networks inherent in biological systems.

## Supporting Information

Table S1 A summary of the numbers and types of loci identified in each of the 932 replicons included in the SIPHT search
(0.29 MB XLS)

Table S2 All predicted loci with homology to previously predicted and/or confirmed ncRNAs in our database
(5.43 MB XLS)

Table S3 All predicted loci lacking homology to but sharing conserved synteny with previously predicted and/or confirmed regulatory RNAs in our database
(2.98 MB XLS)

Table S4 All predicted loci associated with putative binding sites for LexA, sigma-54, or Fur
(0.54 MB XLS)
