# Correction: High-Throughput, Kingdom-Wide Prediction and Annotation of Bacterial Non-Coding RNAs

**DOI:** 10.1371/annotation/a03e1870-1dd7-4c16-8c46-2268eeb2a50a

**Published:** 2008-11-14

**Authors:** Jonathan Livny, Hidayat Teonadi, Miron Livny, Matthew K. Waldor

The published Table S2 is the incorrect version. Please view the correct table here:
